# Nature as the Most Important Coping Strategy Among Cancer Patients: A Swedish Survey

**DOI:** 10.1007/s10943-013-9810-2

**Published:** 2013-12-22

**Authors:** Fereshteh Ahmadi, Nader Ahmadi

**Affiliations:** Faculty of Health and Occupational Studies, University of Gävle, 801 76 Gävle, Sweden

**Keywords:** Religious coping, Spirituality, Nature as a coping method, RCOPE

## Abstract

The authors have conducted a quantitative survey to examine the extent to which the results obtained in a qualitative study among cancer patients in Sweden (Ahmadi, Culture, religion and spirituality in coping: The example of cancer patients in Sweden, Uppsala, Acta Universitatis Upsaliensis, 2006) are applicable to a wider population of cancer patients in this country. In addition to questions relating to the former qualitative study, this survey also references the RCOPE questionnaire (designed by Kenneth I Pargament) in the design of the new quantitative study. In this study, questionnaires were distributed among persons diagnosed with cancer; 2,355 people responded. The results show that nature has been the most important coping method among cancer patients in Sweden. The highest mean value (2.9) is the factor ‘nature has been an important resource to you so that you could deal with your illnesses’. Two out of three respondents (68 %) affirm that this method helped them feel significantly better during or after illness. The second highest average (2.8) is the factor ‘listening to ‘natural music’ (birdsong and the wind)’. Two out of three respondents (66 %) answered that this coping method significantly helped them feel better during illness. The third highest average (2.7) is the factor ‘to walk or engage in any activity outdoors gives you a spiritual sense’. This survey concerning the role of nature as the most important coping method for cancer patients confirms the result obtained from the previous qualitative studies.

## Introduction

Several recent studies have analysed religious- and spirituality-oriented coping methods among secular patients (Ahmadi 2006; Büssing 2010). Yet, most studies on religious- and spiritually oriented coping methods have been conducted among religious persons (Hvidt 2007, 2008; Torbjørnsen et al. 2000; Gall et al. 2000; Alma 1998). However, the research area of religious- and spiritually oriented coping methods rarely considers patients who either are uninterested in institutional religiosity but follow their own individual ways of spirituality or who express no concern for spirituality and religiosity. In order to investigate the presence of religious- and spiritually oriented coping strategies in a mainly secular context, a qualitative study was conducted (Ahmadi 2006) among 51 cancer patients in Sweden where the social majority do not consider themselves religious.^1^ A striking result of this qualitative study concerned nature as a means of spiritual coping. Informants identified nature as an effective factor in dealing with the emotional and psychological problems associated with cancer. Based on the results of this research, a survey study was conducted to examine the extent to which these results might be applicable to a wider population of cancer patients in Sweden. Besides the questions relating to the aforementioned qualitative study, the RCOPE questionnaire designed by Pargament et al. (2000) was referenced when designing the questionnaire for this study. The aim of this survey study was to confirm the previous result concerning the role of the nature as the most important coping strategy among cancer patients in Sweden. This paper presents some results obtained in this survey study.

## Methodology

### Processing

The data were gathered by SKOP, a professional data-gathering agency.^2^ SKOP used several cancer organisations, among them the Swedish Union for Ileostomy-, Colostomy-, and Urostomy-Operated Persons (ILCO); Blood Cancer Association (Blodcancerförbundet) in Stockholm; and the Breast Cancer National Organisation (BRO). Most respondents (61 %) belong to the BRO’s register, a fifth (19 %) belong to Blood Cancer Association’s register, and 20 % belong to register of ILCO. The survey was conducted as a postal questionnaire. No reminder letter was sent out. Data collection was conducted from March to May 2011.

SKOP sent the questionnaires to these cancer organisations, which have access to the names and addresses of their members. The organisations distributed the questionnaires among participants who were chosen randomly by the cancer organisations themselves. Recruiting informants through cancer organisations may bring about certain problems, such as adequate representation. However, given the ethical considerations necessary when dealing with a vulnerable group such as cancer patients, this approach was considered the most suitable for this research.

### Response Rate

In total, about 5,000 questionnaires were distributed among persons diagnosed with cancer in Sweden. Of these, 47 answered that they were not diagnosed themselves but rather were supporting members, 11 were deceased, four had dementia, and 47 questionnaires were returned for unknown reasons. The remaining net sample contained 4,891 persons. A total of 2,417 responses were received, yielding a response rate of 49 %. Despite the seemingly low response rate, this study concludes that this dropout rate is not high considering that similar studies reveal a high dropout rate when studying seriously ill people (Shih 2002).

The authors’ experience shows that cancer patients consider questions relating to existential issues as important. Therefore, they are exceptionally willing to contribute to studies focusing on such issues. Because of ethical considerations, SKOP, the data-gathering agency, chose not to send reminder letters or resend the questionnaire.

### Participants

The study sample consists of 2,417 cancer patients of whom 79 % are women and 21 % are men. Almost one-third (29 %) of those who responded to the survey are 59 years of age or younger; even more (38 %) are between 60 and 69 years of age. One-third (33 %) are 70 years or older. As such, the study consists of three age groups: 18–59 years old, 60–69 years old, and 70+ years old. The reason behind this distribution is that almost a third (29 %) of those who responded to the survey are 59 years old or younger.

The highest proportion of respondents (38 %) are aged between 60 and 69 years, while one-third (33 %) are aged 70 or older.

Regarding the informants’ social class, only a few (4 %) respondents answered that they belong to the upper class and are currently (or were formerly) senior officials, large-scale entrepreneurs, or big farmers. A large majority (64 %) answered that they belong to the middle class and are currently (or were formerly) less-ranking employees, or running small businesses or farms. One-third (32 %) answered that they belong to the working class and are currently (or were formerly) employees.

One-third (33 %) of respondents have attended 9 years or less of school education. Just over one-quarter (28 %) have studied 10–12 years at school. Almost one-fifth (17 %) have 13 years or more of formal education (that is, attended higher education), but have no academic degree. A slightly higher number (21 %) possess an academic degree.

According to Swedish research ethic rules, researchers are forbidden (with the exception of special cases) to pose any questions regarding the informants’ religious affiliation. Therefore, this study lacks information regarding the informants’ religion or faith. However, the study provides information regarding the informants’ belief in a personal God or a higher life-giving force. In response to the question, ‘Do you believe in God?’, less than half (49 %) of the respondents answered in the affirmative. Those who responded that they did not believe in God were asked to answer the question, ‘Do you think there is a higher power or life-giving power?’ Forty-five per cent of those who do not believe in God believe that there is a higher power or a life-giving force. One of the four respondents (25 %) has answered that they follow no particular faith.

## Results and Analysis

### Nature and Coping

The respondents of the present study were asked to choose between twenty-four different factors that they believe helped them feel better when feeling stressed, sad, or depressed during or after their illness. For each factor, the respondents could choose among four alternatives: (1) the factor has helped ‘not at all’; (2) the factor helped to a ‘small’ extent; (3) the factor helped them to a ‘quite large’ extent; or (4) the factor helped them to a ‘very large’ extent. The scale was transformed into a numerical scale with 1 corresponding to ‘not at all’ and 4 corresponding to ‘very large extent’ so that a mean value could be calculated for each factor.

As the results of the study show, the highest mean value (2.9) belongs to the factor indicating that ‘nature’ has been an important resource in dealing with the illness (Table 1). Two out of three respondents (68 %) noted that this factor ‘in a large or quite a large extent’ has helped them feel better when they felt stressed, sad, or depressed during or after the illness. Almost one in three (30 %) answered that nature helped ‘to a very large extent’. Respondents in the younger age category (under 59 years of age) and women have a higher average compared to older persons and men (Table 2).

**Table 1:** When you have felt stressed, sad, or depressed during or after your illness, to what extent have the following helped you feel better?

June 2011	Percentage that answers				Mean	Number of answers
	Not at all (1)	Small (2)	Quite large (3)	Very large (4)		
Thinking about God or contemplating the life of Jesus or other religious personages?	63	20	12	6	1.6	2,266
Thinking about a spiritual power?	49	23	18	9	1.9	2,264
Going to church?	64	21	10	4	1.5	2,261
Praying?	51	20	17	12	1.9	2,320
Listening to religious music?	65	20	11	3	1.5	2,245
Listening to spiritual music?	56	23	16	5	1.7	2,267
Listening to the ‘music of nature’ (birdsong and the whistling of the wind)?	13	21	38	28	2.8	2,335
Going for walks or doing other outdoor activities which give you a sense of spirituality?	19	18	34	29	2.7	2,338
Thinking and contemplating the meaning of life and other things, in solitude?	32	36	26	6	2.1	2,307
Helping others to experience spirituality?	50	28	19	3	1.8	2,282
Thinking that you have done your best and the rest is in God’s hands?	62	18	13	7	1.6	2,310
Praying to God that he will make things better?	58	21	14	6	1.7	2,302
Trying to control your situation without God’s help?	30	20	34	15	2.3	2,283
Trying to stop thinking about your illness by thinking of spiritual matters?	64	25	9	2	1.5	2,327
Seeking spiritual help from a priest or other religious leaders?	86	9	4	2	1.2	2,301
Providing spiritual support to others?	77	16	6	1	1.3	2,290
Having sometime experienced a strong connection to God?	66	16	12	6	1.6	2,324
Having experienced a strong spiritual connection to other people?	57	24	15	4	1.7	2,296
Have you ever believed that your life is part of something greater and higher?	38	27	22	12	2.1	2,323
Having experienced a strong sense of spirituality?	54	23	16	8	1.8	2,314
Believing or feeling that there is a spiritual power within you that helps you cope with your problems?	46	24	20	9	1.9	2,341
Hoping for a spiritual rebirth in this world?	75	17	6	2	1.3	2,285
Preferring to be alone, thinking about your life, or contemplating life, to feel better?	43	32	21	4	1.9	2,304
That nature is an important resource to help you cope with your illness?	11	20	38	30	2.9	2,362

A: Some individuals, but <1 %

**Table 2:** When you have felt stressed, sad, or depressed during or after your illness, to what extent has the following helped you feel better? That nature is an important resource to help you cope with your illness?

	June 2011
Not at all	11
Small	20
Moderate	38
Very great	30
Average	2.9
Number of answers	2,362

Group table	Percentage that answers				Mean	Number of answers
	Not at all (1)	Small (2)	Quite large (3)	Very large (4)		
Age (years)						
−59	9	20	34	37	3.0	694
60–69	9	20	41	30	2.9	913
70	15	21	40	24	2.7	755
Sex						
Female	8	18	39	35	3.0	1,854
Male	21	29	36	14	2.4	503
Social class						
Upper class: higher salaried employees, large business owners/agriculturists	9	22	33	37	3.0	82
Middle class: other salaried employees and business owners/agriculturists	10	20	38	31	2.9	1,490
Working class: workers	13	20	40	27	2.8	743
Place of origin						
City with a population of more than 200 thousand	17	24	33	26	2.7	252
Town with a population of 81–200 thousand	11	23	37	30	2.8	183
Town with a population of 21–80 thousand	10	20	40	30	2.9	464
Community with a population of 20 thousand or less	10	19	39	31	2.9	1,380
Time of diagnosis						
1990 or earlier	11	22	41	27	2.8	329
1991–2000	12	20	37	32	2.9	576
2001–2005	9	19	41	31	2.9	657
2006–2011	11	22	36	31	2.9	759
Organisation						
Blodcancerförbundet	16	23	39	23	2.7	457
BRO	7	18	39	36	3.0	1,450
ILCO	18	25	37	19	2.6	455

The factor with the second highest average (2.8) is ‘listening to the music of nature (such as birdsong and the wind)’ (Table 1). Two out of three respondents (66 %) answer that this factor ‘in a large or quite a large extent’ helped them feel better when during or after illness they felt stressed, sad, or depressed. Just over one in four (28 %) has chosen the alternative ‘to a large extent’. Women and people who grew up in places with 20,000 or fewer residents have higher averages compared to men and those who grew up in larger towns (Table 3).

**Table 3:** When you have felt stressed, sad, or depressed during or after your illness, to what extent has the following made you feel better? Listening to ‘the music of nature’ (birdsong and the whistling of the wind)?

	June 2011
Not at all	13
Small	21
Moderate	38
Very great	28
Average	2.8
Number of answers	2,335

Group table	Percentage that answers				Mean	Number of answers
	Not at all (1)	Small (2)	Quite large (3)	Very large (4)		
Age (years)						
−59	13	22	37	28	2.8	690
60–69	11	20	41	29	2.9	902
70	15	22	36	27	2.7	743
Sex						
Female	10	19	39	33	2.9	1,829
Male	25	30	34	11	2.3	501
Social class						
Upper class: higher salaried employees, large business owners/agriculturists	13	16	39	33	2.9	80
Middle class: other salaried employees and business owners/agriculturists	13	20	38	29	2.8	1,481
Working class: workers	14	24	37	25	2.7	729
Place of origin						
City with a population of more than 200 thousand	19	26	29	26	2.6	250
Town with a population of 81–200 thousand	11	25	39	26	2.8	183
Town with a population of 21–80 thousand	14	21	38	27	2.8	461
Community with a population of 20 thousand or less	12	20	39	29	2.9	1,365
Time of diagnosis						
1990 or earlier	13	24	36	27	2.8	327
1991–2000	11	22	32	35	2.9	568
2001–2005	12	18	43	27	2.9	644
2006–2011	15	21	39	24	2.7	756
Organisation						
Blodcancerförbundet (the Swedish Blood Cancer Association)	20	23	35	22	2.6	457
BRO (The Swedish Breast Cancer Association)	9	18	40	33	3.0	1,434
ILCO (the Swedish Association for Colostomy Patients)	19	28	35	18	2.5	444

The third-placed factor, with an average of 2.7, is ‘to walk or engage in activities outdoors give a spiritual sense’ (Table 1). Three out of five respondents (63 %) answered that this factor has ‘to a very large or quite large extent’ helped them feel better when during or after their illness they felt stressed, sad, or depressed. Almost one in three (29 %) chose the option ‘very large extent’, and one in six (18 %) answered ‘to a small extent’. Women belonging to the upper class and those who grew up in places with 20,000 or fewer inhabitants have higher average than, for instance, men who indicated that they do not belong to the upper class and those who grew up in large cities (Table 4).

**Table 4:** When you have felt stressed, sad, or depressed during or after your illness, to what extent has the following helped you feel better? Going for walks or doing other outdoor activities which give you a sense of spirituality?

	June 2011
Not at all	19
Small	18
Moderate	34
Very great	29
Average	2.7
Number of answers	2,338

Group table	Percentage that answers				Mean	Number of answers
	Not at all (1)	Small (2)	Quite large (3)	Very large (4)		
Age (years)						
−59	18	18	30	34	2.8	690
60–69	16	19	36	29	2.8	903
70	24	19	35	23	2.6	745
Sex						
Female	15	17	35	33	2.9	1,833
Male	33	24	32	11	2.2	500
Social class						
Upper class: higher salaried employees, large business owners/agriculturists	9	19	36	36	3.0	80
Middle class: other salaried employees and business owners/agriculturists	18	18	34	30	2.8	1,477
Working class: workers	23	19	33	25	2.6	734
Place of origin						
City with a population of more than 200 thousand	27	19	30	24	2.5	248
Town with a population of 81–200 thousand	19	21	37	23	2.6	183
Town with a population of 21–80 thousand	18	19	36	28	2.7	461
Community with a population of 20 thousand or less	18	18	34	30	2.8	1,365
Time of diagnosis						
1990 or earlier	26	17	35	23	2.6	325
1991–2000	19	18	32	31	2.7	570
2001–2005	17	18	34	30	2.8	652
2006–2011	19	19	34	28	2.7	749
Organisation						
Blodcancerförbundet	25	20	34	20	2.5	453
BRO	14	17	34	35	2.9	1,434
ILCO	29	22	34	16	2.4	451

### Analysis

The informants of this study regard nature and activities connected to nature as the most significant factors that have helped them to cope with their illness. Therefore, this study hypothesises that the spiritual sanctification of nature is a reason for the choice of nature as a strong coping method among the informants. In fact, this hypothesis is in line with existing theories stating that people sanctify different aspects of life in their search for significance. Such a search becomes more important when facing a serious problem in life. As Pargament (1999:911) pointed out, virtually any object can be perceived as divine-like in character. The sacred qualities can include attributes of transcendence (for example, the holy or heavenly), ultimate value and purpose (for example, that which is blessed or inspiring), and timelessness (for example, the everlasting or the miraculous).

The interpretation of the results of the present study suggests that a large number of informants perceived a sacred value in nature. The timeliness and immensity of nature—the fact that whatever happens in the world, nature will still be there keeping its pace—have had a calming and consoling effect for the severely ill informants in this study. Nature grants a feeling of security when everything else is chaotic. By sanctifying nature as a timeless object, the informants find a spiritual feeling that functions as a therapy in their encounter with cancer.

The prominent position of nature in Swedish ways of thinking and culture might explain why experiences with natural environments play such a central role in coping. In addition to the European Value System Study (EVSS),^3^ other studies such as the Sifo study (Lindén 1994), the Uppsala Study of 1986 (Hamberg 1994), and the 1994 Study (Uddenberg 1995), all indicate that interest in nature and environmental questions is widespread among Swedes, especially among young people. According to the 1994 Study, which was based on a questionnaire survey among 973 Swedes between 20 and 69 years of age, only 4 % replied that ‘we have no need to be out in nature’, while 94 % stressed that ‘nature makes them feel relaxed and harmonic’. In the 1994 Study, 51 % agreed that ‘human beings would feel much better if they were as natural as animals are’.

That informants perceive a sacred value in nature can also be explained by considering the fact that studies have shown that people living in Sweden are spiritual rather than religious. These since church practices and other religious activities have declined drastically in Sweden during the past decades, perhaps even before the mid-twentieth century (Ahmadi 2006; Pettersson and Riis 1994; Sundback 1994; Gustavsoon 1985). Religiosity in Sweden has moved towards a subjective, inwardly directed phenomenon with few public attributes (Sundback 1994:139). Swedes are more likely to describe their religious lives in spiritual terms. Therefore, talk of the existence of a kind of spirituality rather than religiosity is more appropriate among Swedes. This point is observed in this study and in its preceding qualitative investigation (Ahmadi 2006).

## Discussion

The present quantitative study confirms the result obtained in the previous qualitative study concerning the use of nature as a predominant coping method with cancer. As mentioned above, two important tendencies among people in Sweden have presumably influenced their choice of nature as a coping method. These tendencies include seeking spiritual closeness with God or a supreme force and seeking a natural romanticism that makes nature an available source for coping. One factor that may explain the above-mentioned tendencies is the impact of culture and ways of thinking on how individuals deal with their problems. The following will discuss the results obtained in this study by proceeding from a cultural perspective.

### Tendency Towards Spirituality

During the past three centuries, Sweden has moved towards a more individualistic and secular society, where religion has become less organised and more private. This development, together with other characteristics of Swedish culture, such as the romanticisation of nature, has probably given rise to the predominance of spirituality as opposed to religiosity among Swedes. Because Swedes are more likely to describe their religious lives in spiritual terms (Jeffner 1988; Wikström 1998), it seems appropriate to talk about the existence of a kind of spirituality rather than religiosity among Swedes. Concerning this point, thus it is understandable why, for the informants in this study, thinking about spiritual matters and spiritual connection was considered important than religious activities and rituals.

### Tendency Towards Natural Romanticism

Herlitz noted that ‘Swedes generally speaking have an almost sacred relationship to nature’ (1995:36). The post-materialist era witnesses an increased tendency towards ‘private religion’ and spirituality among modern people, especially Swedes. As more of the sacred becomes private, the role of music, literature, psychoanalysis, and nature in mediating existential and ‘religious’ experiences becomes more important. In this regard, nature occupies a special position for Swedes. Modern Swedes seem to seek experiences that used to be mediated by Christian culture, but now in ways other than through traditional religion. One of these alternate ways involves experiencing one’s unity with nature. Being in and feeling a sense of unity with natural environments can give spiritual feelings of unification with the whole of existence. As some informants in the qualitative study stressed, nature becomes the church and unity with the holy becomes unity with nature (Ahmadi 2006).

Such a view of nature signifies a culture in which natural romanticism has been historically strong (Berggren & Trägårdh 2009). The Swedish people generally view themselves as a nature-loving nation (Uddenberg 1995:37). For about a century, the national feelings of Swedish people have been constructed on the basis of, among other things, a profound love of nature (Sundin 1981; Johannisson 1984) that relies on the strong relation between nationalism and the interest in and love of nature in Sweden. Therefore, it is not difficult to understand why many Swedes believe that their well-being depends on having contact with nature (Uddenberg 1995:39) and why such contact is one of the most essential coping methods used by cancer patients in this study.

Based on the results of this study, nature and the possibilities that being in the nature and relating to nature offer cancer patients for coping with their illness should be taken more seriously by health care providers, especially those who deal with the therapy of psychological problem that cancer patients face in different phases, such as diagnosis, treatment, and post-treatment periods. In general, more attention should be devoted to the less conventional therapeutic methods for patients, such as creating possibilities for such patients to come into contact with nature. Well-designed, health-promoting gardens within the clinics, with the possibility for patients to be engaged in gardening, meditation, or just having the chance to feel the earth, in addition to the presence of plants and birdsongs are among examples of using nature to answer the spiritual needs of the patients. This study confirms that these needs are important for people dealing with a serious life crisis.

## Footnotes

1 As Zuckerman (2007) explained, ‘Norris and Inglehart (2004) found that 54 % of Swedes do not believe in God. According to Bondeson (2003), 74 % of Swedes said that they did not believe in “a personal God”. According to Greeley (2003), 46 % of Swedes do not believe in God, although only 17 % self-identify as “atheist”. According to Froese (2001), 69 % of Swedes are either atheist or agnostic. According to Gustafsson and Pettersson (2000), 82 % of Swedes do not believe in a “personal God”. According to Davie (1999), 85 % of Swedes do not believe in ‘God’.

2 See www.skop.se for more information.

3 The European Values Study is a large-scale, cross-national, and longitudinal survey research programme on basic human values, initiated by the European Value Systems Study Group (EVSSG) in the late 1970s, at that time an informal grouping of academics. Now, it is carried on in the setting of a foundation, using the (abbreviated) name of the group European Values Study (EVS).
